# Animation-assisted psychological intervention in pediatric care: mechanisms, clinical applications, and emerging evidence

**DOI:** 10.3389/fpsyg.2026.1969526

**Published:** 2026-10-14

**Authors:** Wu Dongxian, Yu Zeqi, Shu Qi, Wang Shutong

**Affiliations:** 1 Hanseo University, Seosan-si, Republic of Korea; 2 Royal College of Art, London, United Kingdom

**Keywords:** animation, children, dental anxiety, distraction, mental health literacy, preoperative anxiety, procedural pain, virtual reality

## Abstract

Children frequently experience fear, anxiety, and pain during medical encounters, and this distress can lead to poor cooperation, longer procedures, and lasting avoidance of care. Animation, delivered as cartoons, preparation films, applications, serious games, and virtual reality (VR) scenes, has become one of the most widely deployed non-pharmacological adjuncts in pediatric settings because it is inexpensive, non-invasive, and easily standardized. Drawing on a structured search of six databases through July 2026, this mini-review synthesizes randomized and meta-analytic evidence across perioperative anxiety, procedural pain, pediatric dentistry, chronic-disease self-management, and mental health promotion. It organizes the evidence around five psychological pathways: attentional competition, observational learning, exposure and inhibitory learning, narrative-affective processing, and self-efficacy. Additionally, it examines a sixth, non-psychological use in which animation is used to provide therapeutic visual stimulation. Evidence supports moderate short-term benefits for anticipatory anxiety and for needle- and dentistry-related distress, with weaker and less consistent effects for high-intensity procedures and physiological outcomes. We argue that the field’s core issue is conceptual rather than empirical: animation refers to a delivery medium, not a treatment, and trials have compared media rather than mechanisms. This obscures three unresolved problems: discordance between self-reported and autonomic outcomes; an untheorized moderating role of the developmental stage; and a theoretical tension between distraction, which withdraws attention from the feared stimulus, and exposure, which requires engagement with it. Mechanism-matched comparators, core outcome sets, developmentally stratified designs, transparent intervention reporting, and follow-up across repeated procedures are prerequisites for cumulative progress.

## 1 Introduction

Medical encounters are among the most common sources of fear and pain in childhood. Anticipatory anxiety before anesthesia predicts difficult induction, emergence delirium, greater postoperative pain and analgesic consumption, and maladaptive behavior after discharge (Kain et al., 2004; Kain et al., 2006). Needle-related distress reduces procedural cooperation, contributes to the development of persistent needle fear, and is a documented reason for immunization non-compliance in children and adults (Kennedy et al., 2008; McMurtry et al., 2015; Taddio et al., 2012). Dental fear co-occurs with behavior management problems that complicate treatment, and birth-cohort data show childhood anxiety tracking into adulthood with avoidant attendance (Klingberg and Broberg, 2007; Armfield and Heaton, 2013; Thomson et al., 2009). Because pharmacological sedation carries cost, monitoring demands, and adverse-effect risk, non-pharmacological adjuncts have moved to the center of pediatric practice.

Animation differs from other adjuncts in several respects. It is inexpensive, non-invasive, reproducible at negligible marginal cost, and requires little training to deliver; children are already familiar with the medium; and it can be delivered in the induction room, emergency department, dental chair, classroom, or home. The term is now applied to animated distraction videos, animated preparation and psychoeducation films, animated modeling of procedures, animated applications and serious games, and immersive VR content. Trials in each area report benefits, and reviews increasingly treat them as a single intervention class (Oktaviani et al., 2024; Srinivasan et al., 2026).

This aggregation is the problem addressed here. Animation is a medium of delivery not a mechanism of change; categorizing interventions by format obscures differences in the psychological processes they engage and in the outcomes they should be expected to affect. We therefore (i) set out five psychological pathways through which animation plausibly acts, along with one non-psychological use; (ii) summarize the strongest evidence by clinical domain; (iii) review emerging digital formats; and (iv) assess the design, measurement, and reporting problems that limit cumulative inference. The aim is to move the question from whether animation works to asking for whom, under which conditions, and through which pathway it works.

### 1.1 Literature search and selection

The synthesis is narrative rather than systematic. PubMed, Embase, Scopus, Web of Science, PsycINFO, and the Cochrane Library were searched from database inception to 31 July 2026, combining terms for the medium (animation, cartoon, animated video, serious game, and virtual reality), the population (child, pediatric, and adolescent), and emotional, behavioral, or clinical outcomes. Eligible records were English-language randomized trials, quantitative syntheses, and controlled or pilot studies of animated content delivered to patients aged 0–18 years in a health-related setting; pilot and development studies were retained only where they illustrated a mechanism or an emerging format. Foundational theoretical sources and evidence on the consequences of procedural fear were added by citation tracking without a date restriction. Full search strings, eligibility criteria, and the selection flow are reported in the Supplementary material.

## 2 Mechanisms: five psychological pathways and one non-psychological use

### 2.1 Attentional competition

Pain and threat are prepotent claims on a limited-capacity attentional system, and the interruptive function of pain is graded by how completely a competing task occupies that system (Eccleston and Crombez, 1999); gate-control formulations had already allowed for descending, attention-dependent modulation of nociceptive transmission (Melzack and Wall, 1965). Animation competes for attention through motion, color, sound, and narrative suspense while requiring no active response from the child. Meta-analysis supports audiovisual technologies for preoperative anxiety (Oktaviani et al., 2024), and randomized trials show reduced pain and fear during venipuncture using animated video (Düzkaya et al., 2021) and dome-projected VR animation in young children (Lee et al., 2023).

As a mechanistic claim, this generates untested predictions: the benefit should scale with engagement rather than production values, be greatest when nociceptive intensity is low enough to be outcompeted, and dissipate once attention returns to the wound. The burn-dressing literature fits that profile: Animated cartoons reduced pain behavior 5 min after dressing changes but not during them (Feng et al., 2018).

### 2.2 Observational learning

Social cognitive theory holds that children acquire coping behavior by watching models and that vicarious experience is a principal source of self-efficacy (Bandura, 1986). Animated characters are efficient models: they can be designed to resemble the viewer, they show no adverse reaction to the procedure, and they deliver an identical demonstration to every child. Animated modeling extends the Tell-Show-Do tradition in dentistry and has outperformed it on cooperation and anxiety (Sahebalam et al., 2020), as has video modeling more generally (Chinthala et al., 2025). Because this pathway targets learned behavior rather than momentary arousal, its proper outcomes are behavioral, namely cooperation, procedure completion, and attendance, and these should ideally persist to the next appointment, which is rarely assessed.

### 2.3 Exposure and inhibitory learning

Contemporary accounts of exposure emphasize violation of threat expectancies and formation of new inhibitory associations rather than passive habituation (Craske et al., 2014). Animation can present fear cues at controlled intensity inside a benign narrative: The serious game—Dr. Zoo—has children aged 3–6 injecting cartoon animals, and parent-reported needle fear declined during pilot testing (Healy et al., 2023). Additionally, iGET Living pairs animated education with graded exposure for adolescents with chronic pain (Harrison et al., 2026). Critically, this pathway requires the child to focus on the feared stimulus, which is precisely what distraction is designed to prevent.

### 2.4 Narrative and affective processing

Animated narratives supply emotionally legible faces, slowed social situations, and repeatable practice without social cost. The Transporters, which grafted human facial expressions onto vehicles, was built on this logic and improved emotion recognition in autistic children in its original trial (Golan et al., 2010). A Cantonese adaptation reproduced the gain in Hong Kong preschoolers and showed generalization to untaught characters and situations (Chan et al., 2024). The same approach is invoked for empathy training in classrooms (Talu, 2026), body-image micro-interventions (Matheson et al., 2020), and prosocial cartoons that reduce aggression partly by lowering aggressive motivation (Zhang, 2021).

### 2.5 Self-efficacy and empowerment

In chronic illness, animation is used to change behavior over months rather than minutes. Cartoon- and comic-based asthma education improved disease control, quality of life, and school attendance (Akca Sumengen and Ocakci, 2023); animated self-care education outperformed role-play in adolescents with type 1 diabetes (Hosseiniasl et al., 2024); a family-directed animation program raised caregivers’ perioperative knowledge and reduced postoperative nebulization needs after neurosurgery (Lv et al., 2024); and exercise-empowerment games use animated characters to build mastery in children with cancer (Bruggers et al., 2018). In this context, the operative theory is closer to health-behavior and self-regulation models than to attention.

### 2.6 Animation as a carrier of therapeutic stimulation

A conceptually distinct use has emerged in ophthalmology. Dichoptic cartoons present contrast-rebalanced images to the two eyes to overcome suppression, improving acuity faster than patching in children aged 3–5 years in a pilot randomized trial (Jost et al., 2024), with parallel work in intermittent exotropia (Jost et al., 2025) and home-based devices reporting high adherence (Mezad-Koursh et al., 2018). The therapeutic agent is the visual stimulus, not the story; animation contributes to tolerability and compliance. Incorporating such trials into reviews of animation interventions expands the apparent scope of an otherwise psychological literature.

### 2.7 Divergent predictions

These pathways are not variants of one effect. They differ in target (arousal versus learning), timescale (minutes versus months), required attentional stance (disengagement versus engagement), and appropriate outcomes. Trials seldom nominate a pathway in advance. Comparisons of active and passive distraction are therefore best read as manipulations of engagement intensity rather than as tests of animation: an interactive console game outperformed cartoon glasses on self-reported pain but not on anxiety, behavior, or heart rate (Guinot et al., 2021), and cartoon distraction improved cooperation without reducing anxiety or pain during local anesthesia (Üstün et al., 2025). The mechanisms, expected outcomes, and key methodological implications are summarized in Table 1.

**Table 1:** Mechanism-based taxonomy of animation-assisted interventions in pediatric care.

Mechanism	Representative formats and primary target	best-aligned outcomes	Key methodological implication and evidence
Attentional competition	Cartoons, audiovisual videos, tablets, and dome-projected VR. Competes for attention and modulates short-term arousal or pain over minutes.	Pain, fear, cooperation, and procedure duration.	Benefit should scale with engagement and may attenuate when pain is too intense; current evidence is mainly immediate (Eccleston and Crombez, 1999; Melzack and Wall, 1965; Düzkaya et al., 2021; Lee et al., 2023; Feng et al., 2018).
Observational learning	Animated coping models, dental applications, and video modeling. Builds vicarious learning and self-efficacy over one or repeated encounters.	Cooperation, procedure completion, coping behavior, and later attendance.	Use active modeling comparators and assess whether benefits persist to the next appointment (Bandura, 1986; Sahebalam et al., 2020; Chinthala et al., 2025).
Exposure and inhibitory learning	Fear-cue games and graded digital exposure. Promotes threat-expectancy violation and inhibitory learning across repeated sessions.	Fear expectancies, avoidance, and functional engagement.	Requires attention to feared cues and may be undermined by concurrent or habitual distraction (Craske et al., 2014; Healy et al., 2023; Harrison et al., 2026; Blakey and Abramowitz, 2016).
Narrative and affective processing	Emotion-focused stories, social–emotional films, and prosocial cartoons. Supports emotion recognition and affective appraisal over days to weeks.	Emotion recognition, empathy, body image, and aggression.	Assess generalization beyond taught characters and immediate post-exposure ratings (Golan et al., 2010; Chan et al., 2024; Talu, 2026; Matheson et al., 2020; Zhang, 2021).
Self-efficacy and empowerment	Animated self-management education, family programs, and serious games. Builds mastery, self-regulation, and health behavior over weeks to months.	Disease control, quality of life, adherence, and school attendance.	Separate the contribution of animation from added teaching time, structure, and clinician or caregiver contact (Akca Sumengen and Ocakci, 2023; Hosseiniasl et al., 2024; Lv et al., 2024; Bruggers et al., 2018).
Therapeutic visual stimulation (non-psychological)	Dichoptic cartoons and movies. Delivers contrast-rebalanced binocular stimulation through repeated training.	Visual acuity, ocular alignment, and treatment adherence.	Animation improves tolerability, but the therapeutic mechanism is visual stimulation rather than a psychological pathway (Jost et al., 2024; Jost et al., 2025; Mezad-Koursh et al., 2018).

## 3 Evidence across clinical domains

### 3.1 Perioperative anxiety

Preoperative anxiety predicts difficult induction, emergence delirium, greater analgesic requirements, and postoperative behavioral change (Kain et al., 2004; Kain et al., 2006) and is the most studied target. A meta-analysis of audiovisual technologies reports significant reductions in modified Yale Preoperative Anxiety Scale scores before general anesthesia (Oktaviani et al., 2024), and both music and animation lowered pre-induction anxiety and improved induction compliance in a randomized trial in China (Wang et al., 2023). Evidence for home-delivered preparation is weaker: the HIPPO animation video missed its primary endpoint, with only a favorable trend in children aged 7–10 years (Nair et al., 2021), and a caregiver preparation video did not reduce anxiety at induction despite positive caregiver evaluations (Brown et al., 2025). Immersive preoperative education has produced larger effects (Efe et al., 2026), and an animation-based caregiver–child interaction model reduced postoperative pain and perioperative anxiety in preschoolers with congenital heart disease (Chen et al., 2026). Interventions delivered close to the feared event and involving the caregiver as a co-participant have shown greater benefits than take-home content, but these comparisons are observational because timing has not been evaluated as a randomized factor.

### 3.2 Procedural pain in emergency and inpatient settings

Among the 477 children aged 6–12 years in a pediatric emergency unit, the use of an informational animation before intravenous insertion, along with cartoon viewing during the procedure, resulted in lower pain and fear ratings from children, parents, and nurses (Düzkaya et al., 2021). Dome-screen VR animation reduced procedural pain during intravenous placement without requiring a headset (Lee et al., 2023), and tablet distraction showed similar promise in a pilot (Lee et al., 2022). Cartoons improved cooperation and shortened treatment during the suturing of facial lacerations (Choi and Kim, 2021). Additionally, during suture removal, both cartoons and digital games reduced pain, fear, and anxiety, with digital games being more effective (Eroğlu et al., 2026). Effects attenuate as procedural intensity rises: in burn dressing changes, benefits appeared only after the procedure (Feng et al., 2018). Audiovisual distraction has also been compared with physical methods, performing comparably to external cold and vibration in outpatient venipuncture (Bergomi et al., 2018).

### 3.3 Pediatric dentistry

Dentistry has the largest and most internally inconsistent literature. A systematic review and network meta-analysis ranked mobile oral health applications above conventional Tell-Show-Do for anxiety and heart rate, with a Tell-Show-Play-Distraction strategy ranking first, while reporting funnel plot asymmetry (Srinivasan et al., 2026). Immersive VR reduced injection pain compared with non-immersive VR (Kumari et al., 2021), while VR animation reduced dental anxiety in children aged 6–12 years (Mohammadi et al., 2025). In contrast, crossover trials found that cartoon-assisted audiovisual distraction improved behavior and pain perception without reducing anxiety (Üstün et al., 2025) and that visual distraction did not alter anxiety measures (Ghadimi et al., 2018). Heart rate biofeedback was more effective than audiovisual distraction in controlling heart rate during local anesthesia (Padminee et al., 2022), which is an instructive contrast because the intervention that targeted physiological regulation directly was the one that changed a physiological outcome.

### 3.4 Chronic illness and health education

Here the target is behavior rather than momentary affect, and the trials are correspondingly longer and less well controlled. Cartoon-based asthma education improved symptom control, quality of life, and school attendance at 1 and 4 months (Akca Sumengen and Ocakci, 2023), and animated self-care teaching outperformed role-play on quality of life in adolescents with type 1 diabetes (Hosseiniasl et al., 2024); neither trial can separate the animated format from the additional structured teaching time it delivered. Animation also functions as an instructional medium for caregivers and clinicians (Lv et al., 2024). Educational videos improved medical students’ skills in communicating life-limiting diagnoses to parents, with animated and live-action versions performing comparably (Lisakowski et al., 2025), a result worth emphasizing because it suggests that in instructional applications content and structure, rather than animation per se, may carry much of the effect.

### 3.5 Mental health literacy and prevention

This is the fastest-growing area and the one furthest from the distraction paradigm. A teacher-led online mental health literacy program improved high school students’ knowledge and recognition of the need for help (Yamaguchi et al., 2024), and a co-created digital media campaign improved awareness, attitudes, and help-seeking confidence while reducing depression stigma (Curran et al., 2023). Animated anti-bullying literacy resulted in significant knowledge gains among Pakistani primary students (Razzaq et al., 2022), brief animated films improved body satisfaction in children aged 7 to 14 years immediately and at 1 week (Matheson et al., 2020), immersive animation is being tested for child sexual abuse prevention (Rivera-Rivera et al., 2025), and prosocial cartoons have immediate effects on aggression (Zhang, 2021). The limitation of this domain lies in its dependent variables. Knowledge, attitudes, stated intentions, and immediate post-exposure ratings stand as proxies for the outcomes that matter, namely help-seeking, disclosure, and protective behavior, but follow-up rarely extends far enough to observe them. The evidence signals, limitations, and research priorities across clinical domains are summarized in Table 2.

**Table 2:** Clinical evidence, limitations, and research priorities for animation-assisted interventions in pediatric care.

Clinical domain	Evidence signal and representative formats	Main limitations	Priority for future research
Perioperative anxiety	Meta-analytic and randomized evidence supports reduced pre-induction anxiety with audiovisual distraction, immersive education, and caregiver–child models; home preparation alone is less consistent (Oktaviani et al., 2024; Wang et al., 2023; Nair et al., 2021; Brown et al., 2025; Efe et al., 2026; Chen et al., 2026).	Timing and caregiver participation are rarely randomized, and many comparators are minimally engaging.	Factorial tests of timing and caregiver involvement, active comparators, and postoperative and repeated-visit outcomes.
Procedural pain in emergency and inpatient settings	Short-term reductions in pain, fear, and/or improved cooperation are most consistent for venipuncture and suture procedures using information videos, cartoons, dome VR, tablets, or games; effects attenuate in high-intensity burn care (Düzkaya et al., 2021; Lee et al., 2023; Feng et al., 2018; Lee et al., 2022; Choi and Kim, 2021; Eroğlu et al., 2026; Bergomi et al., 2018).	Unblinded ratings, variable procedural intensity, and little follow-up across recurrent procedures, although distress at later procedures is the informative signal (Weisman et al., 1998; Chen et al., 1999).	Define clinically important differences, use mechanism-matched controls, and follow children across repeated procedures.
Pediatric dentistry	Animated modeling, mobile applications, audiovisual distraction, and immersive VR often improve behavior or pain, whereas anxiety and physiological findings remain inconsistent (Srinivasan et al., 2026; Sahebalam et al., 2020; Chinthala et al., 2025; Guinot et al., 2021; Üstün et al., 2025; Kumari et al., 2021; Mohammadi et al., 2025; Ghadimi et al., 2018; Padminee et al., 2022).	Marked intervention and outcome heterogeneity, funnel plot asymmetry, and confounded autonomic measures.	Develop a core outcome set, stratify by developmental stage, and compare directly with active games and biofeedback.
Chronic illness and health education	Asthma and diabetes education, family empowerment, and oncology games show promising improvements in disease control, quality of life, self-care, school attendance, or caregiver knowledge (Akca Sumengen and Ocakci, 2023; Hosseiniasl et al., 2024; Lv et al., 2024; Bruggers et al., 2018).	Small samples and inability to separate animation from additional structured teaching or contact.	Isolate medium from content, measure adherence and behavior, and extend follow-up beyond immediate knowledge or quality-of-life change.
Mental health literacy and prevention	School-based videos, digital campaigns, anti-bullying and body-image films, and immersive prevention commonly improve immediate knowledge, attitudes, stigma, or confidence, but evidence for help-seeking and protective behavior is limited (Matheson et al., 2020; Zhang, 2021; Yamaguchi et al., 2024; Curran et al., 2023; Razzaq et al., 2022; Rivera-Rivera et al., 2025; Cherewick et al., 2021; Ojio et al., 2024).	Proxy outcomes, brief follow-up, and uncertain cultural transfer, scalability, and implementation equity.	Use behavioral endpoints, co-design and culturally adapt content, and test implementation in low-resource settings.

## 4 Emerging formats

Immersive and mixed reality are reshaping delivery. VR replaces an external screen with an enveloping environment, whether through head-mounted displays (Mohammadi et al., 2025) or dome projection that avoids headset tolerance problems in young children (Lee et al., 2023), and immersion appears to matter for pain outcomes (Kumari et al., 2021). Mixed reality overlays virtual elements on the real setting; a shared decision-making game for children with moderate-to-severe atopic dermatitis passed usability testing as a clinical decision aid (Chang et al., 2023).

Applications and serious games add interactivity and agency, from dental anxiety apps (Chinthala et al., 2025) to exposure-based games (Healy et al., 2023), digital graded exposure (Harrison et al., 2026), and empowerment games in oncology (Bruggers et al., 2018). Audio augmentation is a smaller strand: binaural beats added to cartoons lowered heart rate relative to cartoons alone in a preliminary study (Rathi et al., 2024), and animated breathing guidance increased respiratory sinus arrhythmia and reduced arousal in a preregistered field experiment (Obradović et al., 2021), a rare instance of an animation intervention with a specified physiological mechanism. Finally, remote and home delivery extends reach beyond clinical settings through classroom-based online programs (Yamaguchi et al., 2024), home dichoptic training (Mezad-Koursh et al., 2018), distance-learning social–emotional curricula in low-resource settings (Cherewick et al., 2021), and internationally co-developed materials (Ojio et al., 2024).

## 5 Critical appraisal

### 5.1 Comparators

The majority of trials compare animation with treatment-as-usual. Since children, parents, and raters know the allocation, and the experimental arm adds novelty, screen access, and adult attention, estimates confound the putative active ingredient with expectancy and non-specific engagement. This is not hypothetical. Differences shrink when the comparator is itself engaging, as in active versus passive distraction (Guinot et al., 2021), cartoons versus digital games (Eroğlu et al., 2026), and animated versus live-action instruction (Lisakowski et al., 2025). A literature built largely on unblinded comparisons with inert controls cannot establish that animation is itself the active ingredient.

### 5.2 Discordant outcomes

Self-reported anxiety often improves where heart rate or oxygen saturation does not (Üstün et al., 2025; Ghadimi et al., 2018), and occasionally the reverse occurs (Padminee et al., 2022). Three readings compete: response bias in unblinded children; a genuine dissociation between affective and autonomic components of distress; or insensitivity of the physiological measure. The third possibility deserves more weight than it receives, because engaging media are themselves arousing, and intraprocedural heart rate is confounded by crying, movement, and local anesthetic. Heart rate is therefore a poor arbiter of efficacy, yet it is routinely treated as the objective standard against which self-reported improvement is discounted.

### 5.3 Magnitude and interpretation

Samples are typically small, single-center, and briefly followed, conditions that may inflate effect estimates; primary outcomes are often multiple; and minimal clinically important differences are rarely defined. A statistically significant one-point difference on an 11-point pain scale need not correspond to any change in clinical management. The most quantitatively developed area already shows funnel asymmetry (Srinivasan et al., 2026), and almost the entire literature reports positive or neutral results, a distribution that is consistent with selective publication.

### 5.4 Development as an unexamined moderator

Age effects are reported but not theorized. Cartoon distraction during suturing worked best in children aged 3–6 years and less well in younger and older children (Choi and Kim, 2021); preparation videos showed a trend toward benefit only at ages 7–10 years (Nair et al., 2021). A curvilinear pattern is plausible: Symbolic understanding and sustained attention constrain benefit at the lower bound, while content designed for young children loses credibility at the upper bound, where coping shifts toward information and control. Testing this requires developmentally stratified randomization and age-matched content arms rather than *post-hoc* subgroup remarks.

### 5.5 Distraction and exposure make competing demands

The two dominant mechanisms make opposite demands. Distraction succeeds by withdrawing attention from threat; exposure succeeds by directing attention toward it under expectancy-violating conditions (Craske et al., 2014). Habitual distraction may therefore operate as a safety behavior, reducing distress in the moment while preventing the inhibitory learning that would reduce it at the next encounter (Blakey and Abramowitz, 2016). Trials of animation are structurally unable to detect this because almost none follow children into a subsequent procedure. Where the target is recurrent, as in oncology venipuncture, restorative dentistry, or insulin injection, the informative endpoint is distress at the fifth encounter rather than the first. That premise is supported: inadequate analgesia at an initial bone marrow aspiration or lumbar puncture predicted greater pain at subsequent procedures in children with cancer (Weisman et al., 1998), pain memories shape distress at the next procedure (Noel et al., 2012), and reframing those memories reduced anticipatory distress across consecutive lumbar punctures (Chen et al., 1999). Needle fear persists under the daily injection schedule of type 1 diabetes (Cemeroglu et al., 2015). The most recent Cochrane review of needle-procedure interventions, nonetheless, pooled 59 predominantly single-session trials (Birnie et al., 2018).

### 5.6 Intervention reporting

Reports rarely specify duration, timing relative to the procedure, narrative structure, character design, interactivity, or who controls the screen, and dose–response has essentially not been studied. Without intervention descriptions at the level required by contemporary reporting frameworks, the findings cannot be replicated or pooled, and animation remains an unanalyzed bundle. Harms and burdens, including cybersickness, equipment cost and cleaning, workflow disruption, and reinforcement of screen-dependent coping, are seldom recorded.

### 5.7 Culture and equity

Content is disproportionately Western in origin and reused elsewhere through translation. The Cantonese adaptation of the Transporters (Chan et al., 2024) and Japanese–Philippine co-development of mental health videos (Ojio et al., 2024) show that adaptation is feasible and, more importantly, that it is substantive work rather than subtitling. Claims of scalability in low-resource settings (Cherewick et al., 2021) should be checked against device availability, bandwidth, and staff time rather than inferred from low marginal cost. Very large pre–post shifts in knowledge, such as those reported for anti-bullying literacy (Razzaq et al., 2022), also warrant caution, since they may reflect floor effects or test-specific wording rather than meaningful changes in understanding.

## 6 Future directions

Four priorities follow. First, mechanism-first design: trials should nominate a pathway, select the outcomes it predicts, and use mechanism-matched comparators, that is, engaging but mechanistically inert content, with preregistered mediation analysis; second, a core outcome set for pediatric procedural distress specifying instruments, assessment timing, minimal important differences, and at least one behavioral and one longitudinal endpoint; third, developmentally stratified evidence with age-appropriate content arms and prespecified moderator analyses; and fourth, complete intervention reporting, including dose, timing, interactivity, and control of the display.

Two further directions are promising but require caution. Adaptive and personalized delivery, adjusting content in real time to arousal, expression, or behavior, is technically feasible and theoretically attractive, but affective sensing in children raises data-governance and consent questions that should be settled before deployment, and current claims for artificial intelligence in this area exceed the available evidence. Co-design with children, families, and clinicians and genuine cultural adaptation rather than translation are likely to matter more than further technical sophistication (Curran et al., 2023; Ojio et al., 2024).

## 7 Conclusion

Animation has evolved from improvised distraction into a family of structured interventions spanning perioperative anxiety, procedural pain, dentistry, chronic-illness management, mental health promotion, and visual rehabilitation. The evidence supports short-term benefits for anticipatory anxiety and moderate-intensity procedural distress, with effects that vary by setting, age, and outcome, and that attenuate when compared with active comparators. Progress now depends less on new content or hardware than on conceptual discipline: specifying the mechanism a given animation is meant to engage, choosing outcomes and comparators that can test it, and following children beyond the single encounter. Treated as a medium in the service of identifiable psychological processes rather than as an intervention in itself, animation could become a durable component of pediatric care.
