# Supplementary material for: Animation-assisted psychological intervention in pediatric care: mechanisms, clinical applications, and emerging evidence
Source: Front Psychol. 2026 Oct 14;17:1969526. doi: 10.3389/fpsyg.2026.1969526 (PMC13714181; doi:10.3389/fpsyg.2026.1969526)
Supplement: Supplementary file 1 [file Supplementary_file_1.DOCX]

**Supplementary Material**

# S1. Literature search strategy and eligibility criteria

## S1.1 Design

This article is a narrative mini-review supported by a structured and documented literature search. It is not a systematic review: the search was not prospectively registered, screening was not performed in duplicate, formal risk-of-bias appraisal was not applied to every included record, and no quantitative pooling was undertaken. Where pooled estimates are reported in the body of the article, they are taken from published meta-analyses and are attributed as such.

## S1.2 Information sources and search window

Six electronic databases were searched: PubMed/MEDLINE, Embase, Scopus, Web of Science Core Collection, PsycINFO, and the Cochrane Library (CENTRAL and the Cochrane Database of Systematic Reviews). The search covered each database from inception to 31 July 2026. The closing date excludes studies published after that point. Reference lists of all included reviews and trials were hand-searched, and forward citation tracking was performed in Scopus and Google Scholar for the trials carrying the main conclusions.

## S1.3 Search strategy

Three concept blocks were combined with AND: medium, population, and outcome. The PubMed strategy is given in full below and was translated to the syntax and controlled vocabulary of each other database (Emtree terms in Embase, APA thesaurus terms in PsycINFO, topic searches in Scopus and Web of Science).

**Block 1 (medium).** animation[tiab] OR animated[tiab] OR cartoon*[tiab] OR "animated film"[tiab] OR "animated video"[tiab] OR "video modeling"[tiab] OR "video modelling"[tiab] OR "serious game*"[tiab] OR "video game*"[tiab] OR "virtual reality"[tiab] OR "augmented reality"[tiab] OR "mixed reality"[tiab] OR "audiovisual distraction"[tiab] OR "audio-visual distraction"[tiab] OR "mobile application*"[tiab] OR "smartphone app*"[tiab] OR dichoptic[tiab]

**Block 2 (population).** Child[MeSH] OR Adolescent[MeSH] OR Infant[MeSH] OR child*[tiab] OR pediatric*[tiab] OR paediatric*[tiab] OR adolescen*[tiab] OR infant*[tiab] OR preschool*[tiab] OR "young people"[tiab] OR youth[tiab]

**Block 3 (outcome).** anxiety[tiab] OR fear[tiab] OR distress[tiab] OR pain[tiab] OR "dental anxiety"[tiab] OR "preoperative anxiety"[tiab] OR "procedural pain"[tiab] OR cooperation[tiab] OR behaviour*[tiab] OR behavior*[tiab] OR "self-management"[tiab] OR self-care[tiab] OR adherence[tiab] OR "quality of life"[tiab] OR "health literacy"[tiab] OR "mental health"[tiab] OR "emotion recognition"[tiab] OR "visual acuity"[tiab]

**Limits.** English[lang] AND Humans[MeSH]; age filters 0-18 years applied where supported by the database.

## S1.4 Eligibility criteria

**Population.** Children and adolescents aged 0 to 18 years in any health-related context, including perioperative care, emergency and inpatient procedures, pediatric dentistry, outpatient chronic-disease care, ophthalmology, and school-based health promotion. Studies in caregivers or clinicians were eligible only where animated content was the instructional medium under evaluation, and the target of care was a child.

**Intervention.** Animated content as the delivery medium, including two- and three-dimensional animated video, cartoons, animated preparation or psychoeducation films, animated procedural modeling, animated mobile applications and serious games, immersive and non-immersive virtual or mixed reality with animated content, and dichoptic animated stimuli.

**Comparator.** Any comparator, including treatment as usual, an active or alternative distraction condition, another delivery medium, or no comparator in single-arm feasibility work.

**Outcomes.** Anxiety, fear, pain, or distress; cooperation, behavior, procedure completion, or procedure duration; physiological indices such as heart rate, oxygen saturation, or respiratory sinus arrhythmia; disease control, quality of life, adherence, or school attendance; knowledge, attitudes, stigma, or help-seeking; and visual acuity or ocular alignment for the ophthalmological strand.

**Study design.** Randomized controlled trials, including crossover and cluster designs; controlled quasi-experimental studies; and systematic reviews, meta-analyses, and network meta-analyses. Pilot, feasibility, usability, and development studies were retained only where they illustrate a psychological mechanism or an emerging delivery format, and are identified as such in the text.

**Language.** English-language reports only. This restriction is a limitation: relevant work published in Chinese, Japanese, Korean, Turkish, Persian, and other languages will have been missed, and several of the included trials were conducted in settings where local-language publication is common.

**Exclusions.** Live-action or still-image interventions with no animated component; studies in which the animated component could not be distinguished from a wider multicomponent program; studies reporting no separable pediatric data; conference abstracts, editorials, commentaries, letters, and case reports; and preprints or advance online records lacking final publication data.

## S1.5 Second, undated strand: theoretical and background sources

A review organized around mechanisms must cite the founding statements of those mechanisms, and the epidemiological literature establishing why pediatric procedural fear matters. Such sources predate the intervention literature by design and are not retrievable by a search built on the medium. They were therefore identified separately, by targeted lookup and by backward and forward citation tracking from the key trials and reviews, without date restriction. This strand supplied the theoretical sources on gate control, the interruptive function of pain, social cognitive theory, inhibitory learning, and safety behaviors, together with the evidence on the consequences of preoperative anxiety, needle fear, and dental fear, and on distress across repeated procedures. References located by this route are identified in Table S1.

## S1.6 Selection process

Records were screened on title and abstract against the criteria in S1.4, and full texts were retrieved for all records passing that screen. Because the review is narrative, record-level screening counts were not retained, and no PRISMA flow diagram is presented. Selection among eligible records prioritized, in order, quantitative syntheses, randomized trials, controlled studies, and finally pilot or development work cited for illustration. Where several trials supported the same point, preference was given to the largest, the most recent, and those using an active rather than a treatment-as-usual comparator.

# Table S1. References located through the undated targeted strand

| **Ref. no.** | **Reference** |
| --- | --- |
| 1 | Kain ZN, Caldwell-Andrews AA, Maranets I, McClain B, Gaal D, Mayes LC, et al. Preoperative anxiety and emergence delirium and postoperative maladaptive behaviors. Anesth Analg 2004;99(6):1648-1654. doi:10.1213/01.ANE.0000136471.36680.97 |
| 2 | Kain ZN, Mayes LC, Caldwell-Andrews AA, Karas DE, McClain BC. Preoperative anxiety, postoperative pain, and behavioral recovery in young children undergoing surgery. Pediatrics 2006;118(2):651-8. doi:10.1542/peds.2005-2920 |
| 3 | Kennedy RM, Luhmann J, Zempsky WT. Clinical implications of unmanaged needle-insertion pain and distress in children. Pediatrics 2008;122 Suppl 3:S130-3. doi:10.1542/peds.2008-1055e |
| 4 | McMurtry CM, Pillai Riddell R, Taddio A, Racine N, Asmundson GJ, Noel M, et al. Far from "just a poke": common painful needle procedures and the development of needle fear. Clin J Pain 2015;31(10 Suppl):S3-11. doi:10.1097/AJP.0000000000000272 |
| 5 | Taddio A, Ipp M, Thivakaran S, Jamal A, Parikh C, Smart S, et al. Survey of the prevalence of immunization non-compliance due to needle fears in children and adults. Vaccine 2012;30(32):4807-12. doi:10.1016/j.vaccine.2012.05.011 |
| 6 | Klingberg G, Broberg AG. Dental fear/anxiety and dental behaviour management problems in children and adolescents: a review of prevalence and concomitant psychological factors. Int J Paediatr Dent 2007;17(6):391-406. doi:10.1111/j.1365-263X.2007.00872.x |
| 7 | Armfield JM, Heaton LJ. Management of fear and anxiety in the dental clinic: a review. Aust Dent J 2013;58(4):390-407. doi:10.1111/adj.12118 |
| 8 | Thomson WM, Broadbent JM, Locker D, Poulton R. Trajectories of dental anxiety in a birth cohort. Community Dent Oral Epidemiol 2009;37(3):209-19. doi:10.1111/j.1600-0528.2009.00473.x |
| 11 | Eccleston C, Crombez G. Pain demands attention: a cognitive-affective model of the interruptive function of pain. Psychol Bull 1999;125(3):356-366. doi:10.1037/0033-2909.125.3.356 |
| 12 | Melzack R, Wall PD. Pain mechanisms: a new theory. Science 1965;150(3699):971-979. doi:10.1126/science.150.3699.971 |
| 16 | Bandura A. Social foundations of thought and action: a social cognitive theory. Englewood Cliffs, NJ: Prentice-Hall; 1986. |
| 19 | Craske MG, Treanor M, Conway CC, Zbozinek T, Vervliet B. Maximizing exposure therapy: an inhibitory learning approach. Behav Res Ther 2014;58:10-23. doi:10.1016/j.brat.2014.04.006 |
| 59 | Blakey SM, Abramowitz JS. The effects of safety behaviors during exposure therapy for anxiety: critical analysis from an inhibitory learning perspective. Clin Psychol Rev 2016;49:1-15. doi:10.1016/j.cpr.2016.07.002 |
| 60 | Weisman SJ, Bernstein B, Schechter NL. Consequences of inadequate analgesia during painful procedures in children. Arch Pediatr Adolesc Med 1998;152(2):147-9. doi:10.1001/archpedi.152.2.147 |
| 61 | Noel M, Chambers CT, McGrath PJ, Klein RM, Stewart SH. The influence of children's pain memories on subsequent pain experience. Pain 2012;153(8):1563-1572. doi:10.1016/j.pain.2012.02.020 |
| 62 | Chen E, Zeltzer LK, Craske MG, Katz ER. Alteration of memory in the reduction of children's distress during repeated aversive medical procedures. J Consult Clin Psychol 1999;67(4):481-90. doi:10.1037//0022-006x.67.4.481 |
| 63 | Cemeroglu AP, Can A, Davis AT, Cemeroglu O, Kleis L, Daniel MS, et al. Fear of needles in children with type 1 diabetes mellitus on multiple daily injections and continuous subcutaneous insulin infusion. Endocr Pract 2015;21(1):46-53. doi:10.4158/EP14252.OR |
| 64 | Birnie KA, Noel M, Chambers CT, Uman LS, Parker JA. Psychological interventions for needle-related procedural pain and distress in children and adolescents. Cochrane Database Syst Rev 2018;10(10):CD005179. doi:10.1002/14651858.CD005179.pub4 |

*Reference numbers correspond to the reference list of the revised manuscript.*
